# Supplementary material for: Drought-exposure history increases complementarity between plant species in response to a subsequent drought
Source: Nat Commun. 2022 Jun 9;13:3217. doi: 10.1038/s41467-022-30954-9 (PMC9184649; doi:10.1038/s41467-022-30954-9)
Supplement: Supplementary file 1 — Supplementary Information [file 41467_2022_30954_MOESM1_ESM.pdf]

**SUPPLEMENTARY INFORMATION FOR**

# Drought-exposure history increases complementarity between plant species in response to a subsequent drought

**Authors:** Yuxin Chen, Anja Vogel, Cameron Wagg, Tianyang Xu, Maitane Iturrate Garcia, Michael Scherer-Lorenzen, Alexandra Weigelt, Nico Eisenhauer, Bernhard Schmid

***Correspondence**: yuxin.chen@xmu.edu.cn and bernhard.schmid@uzh.ch

## SUPPLEMENTARY METHODS

### Experimental design in the glasshouse

We collected seeds of 17 species in total but subsequently discarded two species with few seeds. We germinated the other 15 species, which included 6 short herbs, 3 tall herbs, 3 legumes and 3 grasses (see reference 1 for definition of these four functional groups). We designed the species pairs in the glasshouse based on these four functional groups (FGs) of the 15 species using the following criteria. (1) Species pairs within each FG: for the FGs with three species (tall herb, legume and grass), we used all the pairwise combinations (n=3); for the FG with six species (short herb), we created six pairs, in which each species occurred twice across the pairs. (2) Species pairs between FGs: for the combinations without short herbs, we randomly created three species pairs; for the combinations with short herbs, we randomly created 6 species pairs, in which species from short herbs occurred once across the pairs, and species from other FGs occurred twice across the pairs. This design yielded 42 species pairs in total. But after the germination, we found that three out of the 15 species had very low germination rates and few seedlings. Thus, we only used the 12 species with enough seedlings for the biodiversity experiment in the glasshouse, which resulted in 25 possible species pairs. Of these, we had to discard four species pairs due to mortality during seedling transplantation, which resulted in the final number of 21 species pairs.

Next, we considered the two types of treatment histories in the Jena Experiment: drought and biodiversity. We considered the biodiversity history at the level of FG instead of species, because trait differences between species were much larger between than within FGs [1], and manipulating history combinations of drought x FG richness allowed a more balanced design with larger potential effects than having

history combinations of drought x species richness (which would also have been difficult due to limitations of space and labor resources). To separate the effects of drought selection history from that of biodiversity selection history, plants originating from Jena subplots with the same FG composition were combined for each drought selection history. With the above criteria and after exclusions of failed establishment, we obtained 257 pots of mixtures.

**Supplementary Table 1 | Species information and number of replicates of species composition (identity of species pair) before the drought event in the glasshouse.** Numbers in brackets indicate the numbers of replicates for ambient- vs. drought-selected plants, respectively. Data in the diagonal are for monocultures (in blue, planted in blocks 1–4); data in the lower diagonal are for 2-species mixtures (in red, planted in blocks 1–4). The last two rows are for individuals from pots with one individual planted in blocks 1–4 and 5 (in orange), respectively. Upper-case letters indicate functional groups (G=grass, S=short herb, T=tall herb, L=legume). All species were perennial except for *Trifolium dubium*.

| Species full name | Short name | AP1 (G) | AP2 (G) | BP (S) | CB (T) | LC (L) | PL (S) | PM (S) | PT (G) | PV (S) | RA (T) | TO (S) | TD (L) |
|---|---|---|---|---|---|---|---|---|---|---|---|---|---|
| *Alopecurus pratensis* | AP1 | (11, 11) | -- | -- | -- | -- | -- | -- | -- | -- | -- | -- | -- |
| *Avenula pubescens* | AP2 | (8, 7) | (9, 9) | -- | -- | -- | -- | -- | -- | -- | -- | -- | -- |
| *Bellis perennis* | BP | (10, 10) | -- | (14, 14) | -- | -- | -- | -- | -- | -- | -- | -- | -- |
| *Crepis biennis* | CB | (1, 1) | -- | -- | (2, 2) | -- | -- | -- | -- | -- | -- | -- | -- |
| *Lotus corniculatus* | LC | -- | -- | -- | -- | (1, 1) | -- | -- | -- | -- | -- | -- | -- |
| *Plantago lanceolata* | PL | (8, 8) | -- | -- | (1, 1) | -- | (8, 7) | -- | -- | -- | -- | -- | -- |
| *Plantago media* | PM | -- | -- | -- | (1, 1) | -- | -- | (16, 16) | -- | -- | -- | -- | -- |
| *Poa trivialis* | PT | (11, 10) | (7, 7) | -- | -- | -- | -- | (16, 15) | (15, 15) | -- | -- | -- | -- |
| *Prunella vulgaris* | PV | -- | (4, 4) | (8, 8) | -- | -- | (7, 7) | -- | -- | (8, 8) | -- | -- | -- |
| *Rumex acetosa* | RA | -- | (5, 6) | (12, 12) | -- | (1, 1) | -- | -- | -- | (8, 8) | (14, 14) | -- | -- |
| *Taraxacum officinale* | TO | -- | -- | -- | -- | -- | (4, 4) | (3, 3) | (4, 4) | -- | -- | (4, 4) | -- |
| *Trifolium dubium* | TD | -- | -- | -- | -- | -- | (5, 5) | -- | (6, 5) | -- | -- | -- | (7, 7) |
| Individual (blocks 1–4) | | (11, 11) | (9, 9) | (14, 14) | (2, 2) | (2, 3) | (7, 8) | (16, 16) | (15, 15) | (8, 8) | (14, 14) | (6, 5) | (5, 5) |
| Individual (block 5) | | (22, 16) | (12, 13) | (13, 15) | (5, 4) | (2, 2) | (11, 11) | (14, 13) | (17, 15) | (4, 4) | (8, 11) | (3,3) | (12, 11) |

**Supplementary Table 2 | Spearman's rank correlation of biodiversity effects on productivity between different harvests, separately for ambient- and drought-selected plants.** Biodiversity effects were calculated as net effect (NE, in grey), complementarity effect (CE, in light orange) and sampling effect (SE, in light blue). T1–3 represent harvests before, during and after the drought event in the glasshouse. For each biodiversity effect of each selection treatment, the above triangle shows the *P* values (based on two-sided tests); the below triangle shows the correlation coefficients. Numbers within brackets represent the number of species pairs. Data in bold represent significant results ($P < 0.05$).

| | | Ambient-selected plants | | | Drought-selected plants | | |
|---|---|---|---|---|---|---|---|
| NE | | | | | | | |
| | | T1 (21) | T2 (21) | T3 (20) | T1 (21) | T2 (20) | T3 (19) |
| | T1 | | **0.022** | 0.431 | | **0.043** | 0.556 |
| | T2 | **0.503** | | 0.828 | **0.459** | | 0.431 |
| | T3 | -0.187 | -0.052 | | -0.144 | 0.191 | |
| CE | | | | | | | |
| | | T1 (21) | T2 (21) | T3 (15) | T1 (20) | T2 (20) | T3 (17) |
| | T1 | | **0.009** | 0.743 | | **0.015** | **0.002** |
| | T2 | **0.560** | | 0.903 | **0.540** | | 0.737 |
| | T3 | 0.093 | 0.036 | | **-0.699** | -0.088 | |
| SE | | | | | | | |
| | | T1 (21) | T2 (21) | T3 (15) | T1 (20) | T2 (20) | T3 (17) |
| | T1 | | **0.006** | 0.532 | | 0.245 | 0.352 |
| | T2 | **0.591** | | 0.490 | 0.272 | | 0.891 |
| | T3 | 0.175 | -0.193 | | -0.240 | -0.037 | |

**Supplementary Table 3 | Significance tests against zero for net biodiversity effect (NE), complementarity effect (CE) and sampling effect (SE) before, during and after the drought event in the glasshouse for the two selection treatments.** Results are from mixed-effects analyses of variance by fitting block and species composition as fixed- and random-effects terms, respectively. Data in bold represent significant results (*P* < 0.05).

| | Ambient-selected plants | | | | Drought-selected plants | | | |
|---|---|---|---|---|---|---|---|---|
| | *df* | *ddf* | *F* | *P* | *df* | *ddf* | *F* | *P* |
| **Before drought** | | | | | | | | |
| NE (127, 128) | 1 | 17.3 | 4.540 | **0.048 +** | 1 | 16.6 | 4.798 | **0.043 +** |
| CE (126, 128) | 1 | 13.8 | 1.675 | 0.217 + | 1 | 14.4 | 1.653 | 0.219 + |
| SE (126, 128) | 1 | 18.7 | 3.201 | 0.090 + | 1 | 17.4 | 9.429 | **0.007 +** |
| **During drought** | | | | | | | | |
| NE (126, 128) | 1 | 17.4 | 0.149 | 0.705 – | 1 | 18.6 | 2.309 | 0.145 – |
| CE (124, 124) | 1 | 20.0 | 0.070 | 0.788 – | 1 | 16.9 | 5.985 | **0.026 –** |
| SE (124, 124) | 1 | 19.6 | 0.051 | 0.824 – | 1 | 18.4 | 0.013 | 0.910 – |
| **After drought** | | | | | | | | |
| NE (109, 110) | 1 | 13.4 | 0.022 | 0.882 – | 1 | 16.2 | 10.330 | **0.005 +** |
| CE (97, 91) | 1 | 9.9 | <0.001 | 0.993 – | 1 | 13.4 | 21.480 | **<0.001 +** |
| SE (97, 91) | 1 | 10.9 | 0.867 | 0.372 – | 1 | 14.6 | 14.690 | **0.002 –** |

*df*, numerator degrees of freedom; *ddf*, denominator degrees of freedom (these reflect residual degrees of freedom among the 15–21 species pairs [= species compositions] for which biodiversity effects were calculated). *F* and *P* indicate *F* ratios and *P* values of the significance tests, respectively. + or – besides the *P* values represents the direction of effects. Numbers within brackets indicate the numbers of pots for plants

under ambient or drought conditions in the glasshouse, respectively.

**Supplementary Table 4 | Significance tests for the effects of selection treatment on stability calculated separately for mixtures and monocultures**. Results are from mixed-effects analyses of variance by fitting block and selection treatment as fixed-effects terms, species composition and its interaction with selection treatment as random-effects terms. Data in bold represent significant results (*P* < 0.05).

| | *df* | *ddf* | *F* | *P* |
|---|---|---|---|---|
| **Resistance** | | | | |
| Mixture (255) | 1 | 15.4 | 1.690 | 0.212 – |
| Monoculture (209) | 1 | 5.1 | 0.668 | 0.450 + |
| **Recovery** | | | | |
| Mixture (255) | 1 | 6.9 | 7.388 | **0.030 +** |
| Monoculture (206) | 1 | 9.8 | 0.044 | 0.839 – |
| **Resilience** | | | | |
| Mixture (255) | 1 | 15.8 | 1.020 | 0.328 + |
| Monoculture (209) | 1 | 10.2 | 0.003 | 0.955 + |

*df,* numerator degrees of freedom; *ddf*, denominator degrees of freedom (these reflect residual degrees of freedom across species compositions). *F* and *P* indicate *F* ratios and the *P* values of the significance tests, respectively. + or – besides the *P* values represents the direction of difference between drought vs. ambient selection treatments. Numbers within brackets indicate the numbers of pots.

**Supplementary Table 5 | Significance tests for the effects of selection treatment on the difference in stabilities between mixtures and monocultures.** Results are from mixed-effects analyses of variance by fitting block and selection treatment as fixed-effects terms, species composition and its interaction with selection treatment as random-effects terms. Data in bold represent significant results ($P < 0.05$).

| | *df* | *ddf* | *F* | *P* |
|---|---|---|---|---|
| Resistance (254) | 1 | 16.5 | 4.627 | **0.047** – |
| Recovery (248) | 1 | 19.0 | 6.550 | **0.020 +** |
| Resilience (254) | 1 | 14.7 | 0.686 | 0.421 + |

*df*, numerator degrees of freedom; *ddf*, denominator degrees of freedom (these reflect residual degrees of freedom across species compositions). *F* and *P* indicate *F* ratios and the *P* values of the significance tests, respectively. + or – besides the *P* values represents the direction of difference between drought vs. ambient selection treatments. Numbers within brackets indicate the numbers of mixtures.

**Supplementary Table 6 | Significance tests for the effects of selection treatment on neighbor interaction intensity in mixtures and monocultures before, during and after the drought event in the glasshouse.** Results are from mixed-effects analyses of variance by fitting block and selection treatment as fixed-effects terms, species composition and its interaction with selection treatment as random-effects terms. Data in bold represent significant results ($P < 0.05$).

| | *df* | *ddf* | *F* | *P* |
|---|---|---|---|---|
| **Before drought** | | | | |
| Mixture (257) | 1 | 11.2 | 0.037 | 0.851 – |
| Monoculture (216) | 1 | 10.4 | 0.639 | 0.442 – |
| **During drought** | | | | |
| Mixture (253) | 1 | 14.1 | 6.330 | **0.025 –** |
| Monoculture (205) | 1 | 9.7 | 1.710 | 0.222 – |
| **After drought** | | | | |
| Mixture (245) | 1 | 9.3 | 0.245 | 0.632 + |
| Monoculture (195) | 1 | 5.2 | 0.140 | 0.720 – |

*df,* numerator degrees of freedom; *ddf*, denominator degrees of freedom (these reflect residual degrees of freedom across species compositions). *F* and *P* indicate *F* ratios and the *P* values of the significance tests, respectively. + or – besides the *P* values represents the direction of difference between drought vs. ambient selection treatments. Numbers within brackets indicate the numbers of pots.

**Supplementary Table 7 | Significance tests for the difference between heterospecific and conspecific interactions before, during and after the drought event in the glasshouse for the two selection treatments.** Results are from mixed-effects analyses of variance by fitting block and species composition as fixed- and random-effects term, respectively. Data in bold represent significant results ($P < 0.05$).

| | *df* | *ddf* | *F* | *P* |
|---|---|---|---|---|
| **Before drought** | | | | |
| Drought (127) | 1 | 14.9 | 0.158 | 0.696 + |
| Ambient (130) | 1 | 6.3 | 2.348 | 0.174 – |
| **During drought** | | | | |
| Drought (125) | 1 | 14.2 | 0.635 | 0.439 – |
| Ambient (128) | 1 | 17.7 | 1.963 | 0.178 + |
| **After drought** | | | | |
| Drought (120) | 1 | 15.6 | 22.680 | **<0.001** + |
| Ambient (123) | 1 | 13.8 | 4.495 | 0.053 + |

*df*, numerator degrees of freedom; *ddf*, denominator degrees of freedom (these reflect residual degrees of freedom across species compositions). *F* and *P* indicate *F* ratios and the *P* values of the significance tests, respectively. + or – besides the *P* values represents the direction of difference between heterospecific vs. conspecific interaction. Numbers within brackets indicate the numbers of mixtures.

**Supplementary Table 8 | Significance tests for the effects of selection treatment on the difference between heterospecific and conspecific interaction before, during and after the drought event in the glasshouse.** Results are from mixed-effects analyses of variance by fitting block and selection treatment as fixed-effects terms, species composition and its interaction with selection treatment as random-effects terms. Data in bold represent significant results ($P < 0.05$).

| | *df* | *ddf* | *F* | *P* |
|---|---|---|---|---|
| Before drought (n=257) | 1 | 9.1 | 1.985 | 0.192 + |
| During drought (n=253) | 1 | 15.4 | 2.677 | 0.122 – |
| After drought (n=243) | 1 | 10.1 | 10.640 | **0.008 +** |

*df*, numerator degrees of freedom; *ddf*, denominator degrees of freedom (these reflect residual degrees of freedom across species compositions). *F* and *P* indicate *F* ratios and the *P* values of the significance test, respectively. + or – besides the *P* values represents the direction of difference between drought vs. ambient selection treatments. Numbers within brackets indicate the numbers of mixtures.

**Supplementary Table 9 | Significance tests for the effects of selection treatment on traits measured on individual plants without neighbors.** Results are from analyses of variance with general linear models. Numbers within brackets indicate the numbers of individual plants. Data in bold represent significant results (*P* < 0.05).

| | *df* | *P* |
|---|---|---|
| **Leaf relative chlorophyll content before drought (n=420)** | | |
| Species | 11 | <0.001 |
| Selection treatment | 1 | 0.847 |
| Species x selection treatment | 11 | 0.175 |
| **Leaf area before drought (n=422)** | | |
| Species | 11 | <0.001 |
| Selection treatment | 1 | 0.257 |
| Species x selection treatment | 11 | 0.400 |
| **Leaf mass per area before drought (n=422)** | | |
| Species | 11 | <0.001 |
| Selection treatment | 1 | 0.330 |
| Species x selection treatment | 11 | **0.019** |
| **Leaf osmometric potential before drought (n=359)** | | |
| Species | 11 | <0.001 |
| Selection treatment | 1 | 0.973 |
| Species x selection treatment | 10 | 0.506 |
| **Leaf stomatal conductance before drought (n=329)** | | |
| Species | 11 | <0.001 |
| Selection treatment | 1 | 0.500 |

| | | |
|---|---:|---:|
| Species x selection treatment | 11 | 0.886 |
| **Leaf stomatal conductance during drought (n=152)** | | |
| Species | 10 | 0.012 |
| Selection treatment | 1 | 0.454 |
| Species x selection treatment | 10 | 0.974 |
| **Root-shoot biomass ratio after drought (n=368)** | | |
| Species | 11 | <0.001 |
| Selection treatment | 1 | 0.526 |
| Species x selection treatment | 11 | 0.488 |

*df* and *P* indicate numerator degrees of freedom and the *P* values of the significance test, respectively.

**Supplementary Table 10 | Significance tests for the effects of selection treatment on trait dissimilarity between interacting species in mixtures.** Results are from mixed-effects analyses of variance by fitting block and selection treatment as fixed-effects terms, species composition and its interaction with selection treatment as random-effects terms. Traits were measured on plants within mixtures. Data in bold represent significant results ($P < 0.05$).

| | *df* | *ddf* | *F* | *P* |
|---|---|---|---|---|
| Leaf relative chlorophyll content (n=237) | 1 | 14.7 | 0.912 | 0.355 + |
| Leaf area (n=232) | 1 | 19.0 | 3.660 | **0.071 +** |
| Leaf mass per area (n=232) | 1 | 12.0 | 0.041 | 0.843 – |
| Three traits (n=230) | 1 | 12.3 | 1.483 | 0.246 + |

*df*, numerator degrees of freedom; *ddf*, denominator degrees of freedom (these reflect residual degrees of freedom across species composition). *F* and *P* indicate *F* ratios and the *P* values of the significance test, respectively. + or – besides the *P* values represents the direction of difference between drought vs. ambient selection treatments. Numbers within brackets indicate the numbers of mixtures.

## SUPPLEMENTARY FIGURES

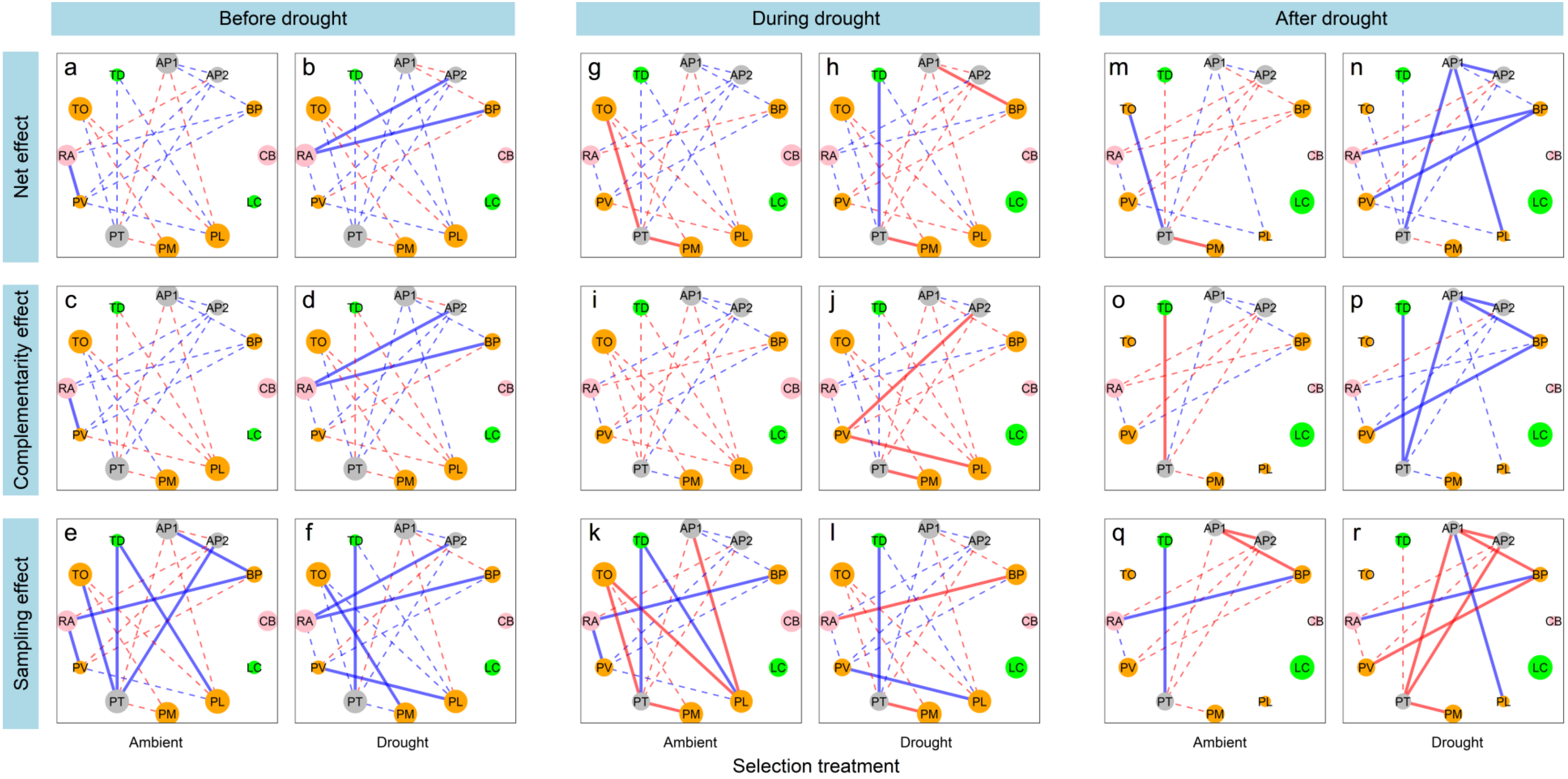


**Supplementary Figure 1 | Effects of biodiversity on productivity among different 2-species mixtures for each selection treatment**

**(ambient- vs. drought-selected plants) and harvest (before [a–f], during [g–l] and after [m–r] the drought event in the glasshouse).** Biodiversity effects were calculated as net effect (first row), complementarity effect (second row) and sampling effect (third row). Lines connecting nodes (species) indicate species pairs within mixtures in the glasshouse. Blue and red lines indicate positive and negative average biodiversity effects, respectively, with the solid lines indicating significant effects based on Student's t test ($\alpha = 0.05$, two-sided tests; mixtures of each selection treatment with less than three replicates at a harvest were not tested for significance and hence not shown in this figure). Node sizes are proportional to the corresponding monoculture biomass in each panel. Node colors indicate different functional groups (green=legume, grey=grass, orange=short herb, pink=tall herb). Full species names are shown in Supplementary Table 1. Source data are provided as a Source Data file. The numbers of mixtures per species pair are provided in the Source Data file.

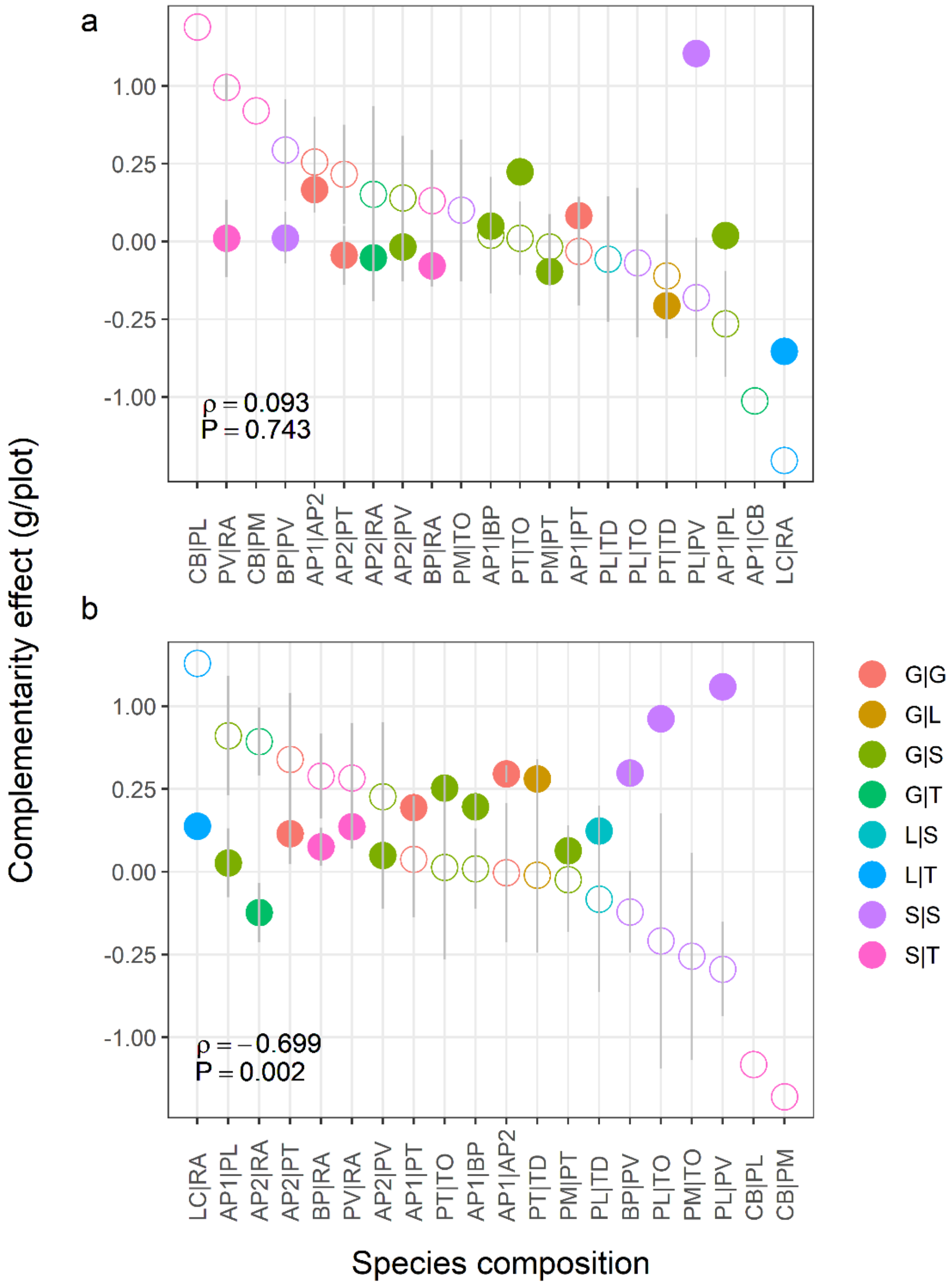


**Supplementary Figure 2 | Relationships between complementarity effects (CE) before (empty circles) vs. after (filled circles) the drought event in the glasshouse.** Panels a and b show the CEs from ambient and drought selection treatments in the field, respectively. The horizontal axes show the species pairs, which were ranked according to their CEs before the drought event in the glasshouse. The circles and

lines show the mean ± standard error of CEs for each species pair. Different colors represent different functional group compositions (G = grass, L = legume, S = short herb and T = tall herb). The equations show the Spearman's rank correlation coefficients and *P* values based on two-sided tests. The error bars of species pairs with less than three replicates are not shown. Full species names are shown in Supplementary Table 1. Source data are provided as a Source Data file. The numbers of mixtures per species pair are provided in the Source Data file.

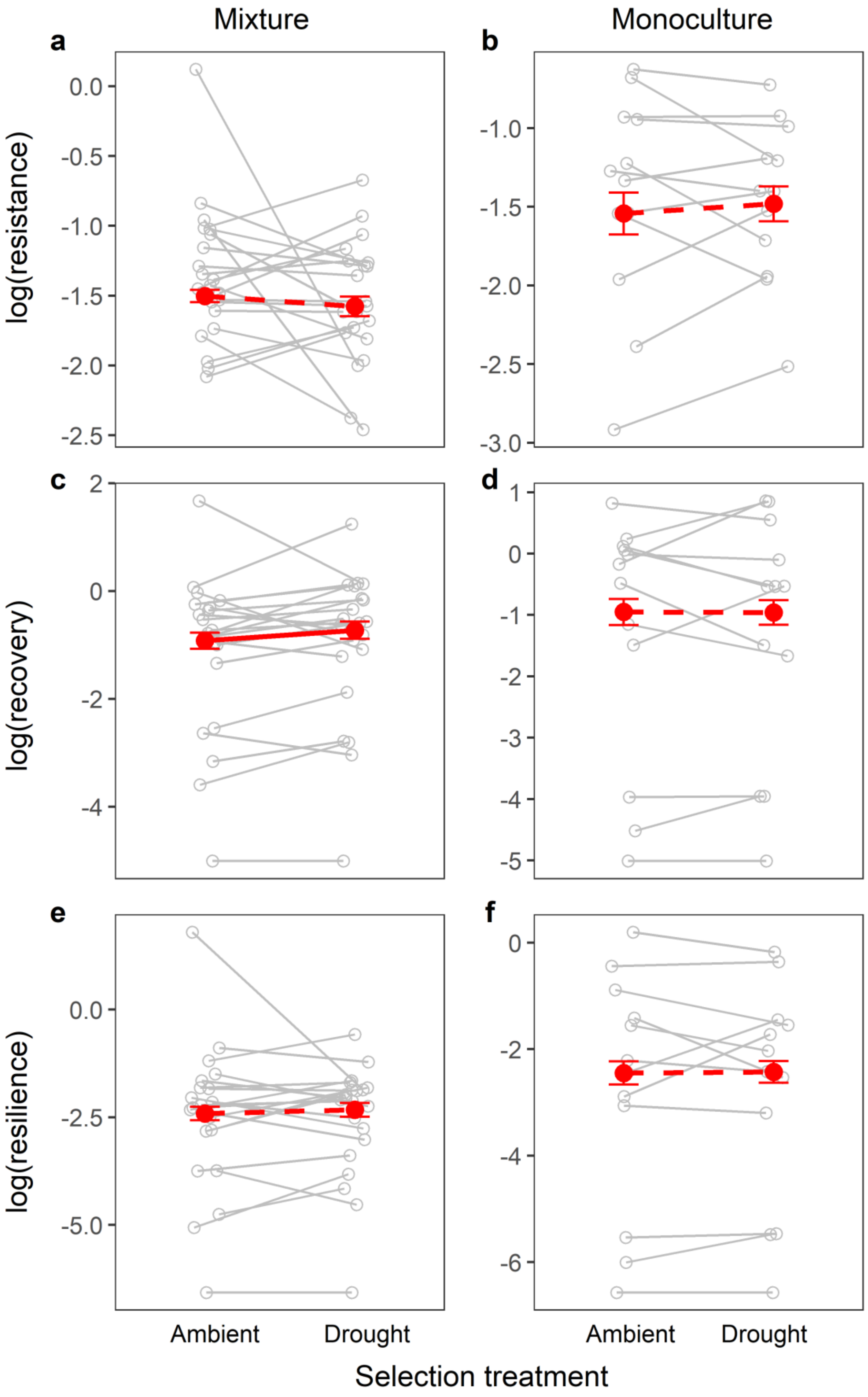


**Supplementary Figure 3 | Difference in biomass stabilities (resistance [a–b],**

**recovery [c–d] and resilience [e–f]) in response to the drought event in the glasshouse between selection treatments (plants selected under ambient vs. drought conditions), calculated separately for mixtures (first column) and monocultures (second column).** The solid red line indicates a significant difference between the two selection treatments ($P < 0.05$ in mixed-model analysis of variance, see Supplementary Table 4). Red points and error bars show means ± standard error. Grey points represent means for species pairs (standard errors for species pairs were not shown). Grey lines connect the two selection treatments for each species pair. Source data are provided as a Source Data file. The numbers of mixtures per species pair and per selection treatment are provided in the Source Data file.

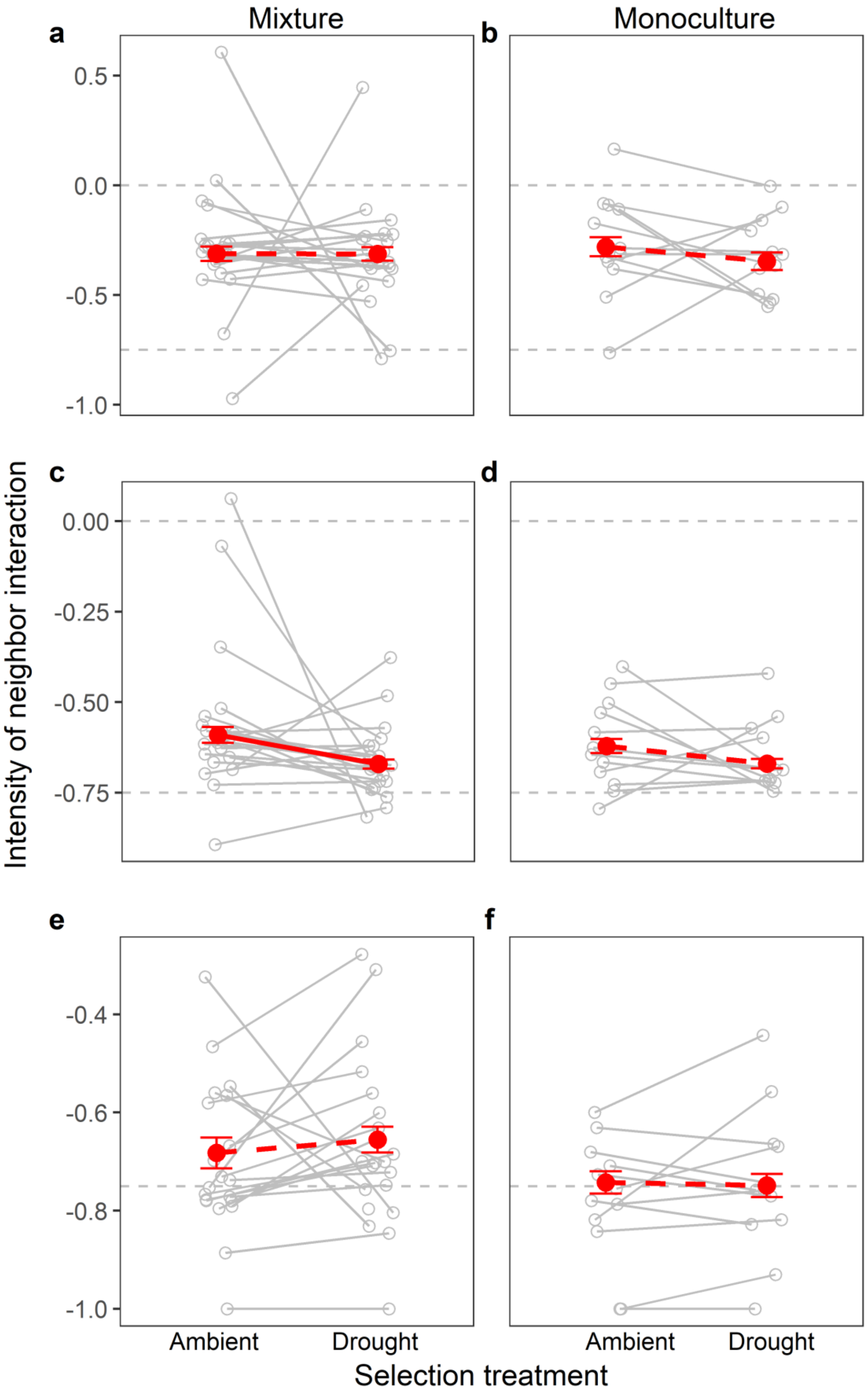


**Supplementary Figure 4 | Effects of selection treatment (ambient- vs. drought-**

**selected plants) on neighbor interaction intensity for 2-species mixtures (first column) and monocultures (second column) before (a–b), during (c–d) and after (e–f) the drought event in the glasshouse.** The solid red line indicates a significant difference between the two selection treatments ($P < 0.05$ in mixed-model analysis of variance, see Supplementary Table 6). Red points and error bars show means ± standard error. Grey points represent means for species pairs (standard errors for species pairs were not shown). Grey solid lines connect the same species pair between the two selection treatments for each species pair. Grey dashed lines at zero indicate the case when individual plants with vs. without neighbors have the same biomass. Grey dashed lines at –0.75 indicate the case when an individual grown alone in a pot is four times bigger than an individual grown in a pot with four individuals. Source data are provided as a Source Data file. The numbers of mixtures per species pair and per selection treatment are provided in the Source Data file.

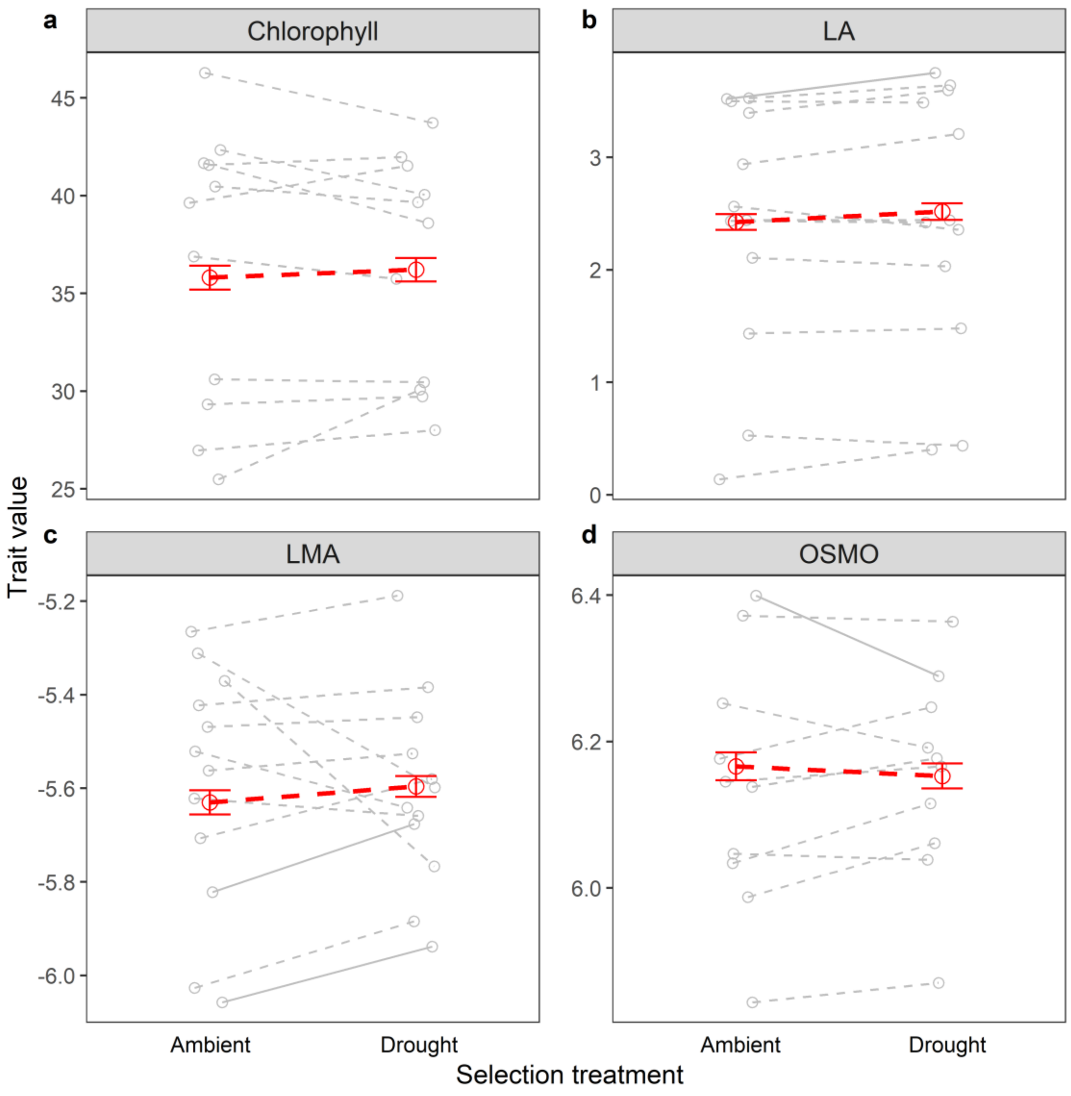


**Supplementary Figure 5 | Effects of selection treatment (ambient- vs. drought-selected plants) on trait values (leaf relative chlorophyll content [a], leaf area [b, LA], leaf mass per area [c, LMA] and leaf osmometric potential [d, OSMO]) measured on individual plants before the drought event in the glasshouse.** Red points and error bars show means ± standard error of all individual plants. Grey points represent means for species (standard errors for species were not shown). Grey lines connected the same species between the selection treatments. Solid lines represent statistically significant difference ($P < 0.05$) in trait values between the two selection treatments. The statistical tests for all species together were conducted by fitting block and selection treatment as fixed-effects terms, species composition and

its interaction with selection treatment as random-effects terms in mixed-effects models ($P > 0.10$). The statistical tests for each species were conducted by fitting block and selection treatment in general linear models. Note that the values of LA, LMA and OSMO were log-transformed. Source data are provided as a Source Data file. The numbers of pots per species and per selection treatment, and the results of statistical tests for each species separately and all species jointly are provided in the Source Data file.

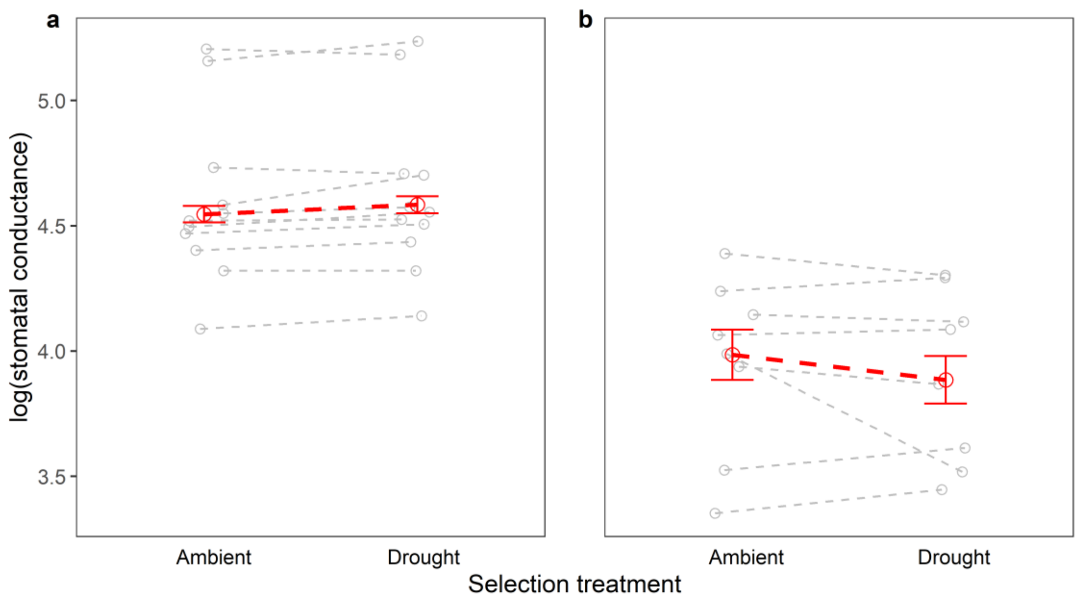


**Supplementary Figure 6 | Effects of selection treatment (ambient- vs. drought-selected plants) on leaf stomatal conductance measured on individual plants before (a) and during (b) the drought event in the glasshouse.** Red points and error bars show means $\pm$ standard error of all individual plants. Grey points represent means for species (standard errors for species were not shown). Grey lines connected the same species between the selection treatments. Dashed lines represent statistically insignificant difference ($P > 0.10$) in trait values between the two selection treatments. The statistical tests for all species together were conducted by fitting selection treatment as fixed-effects terms, species composition and its interaction with selection treatment as random-effects terms in mixed-effects models. The statistical tests for each species were conducted by fitting selection treatment in general linear models. Block was additionally included as fixed-effect term in panel a. Source data are provided as a Source Data file. The numbers of pots per species and per selection treatment, and the results of statistical tests for each species separately and all species jointly are provided in the Source Data file.

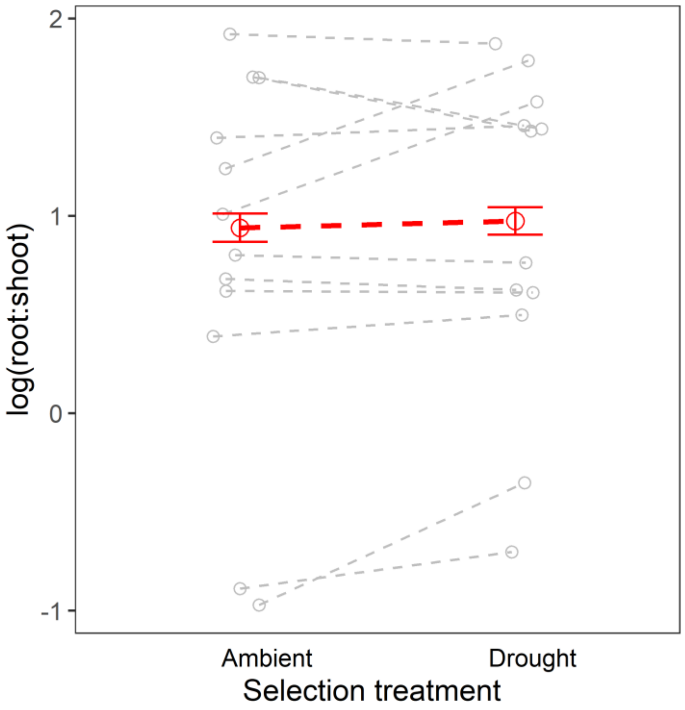


**Supplementary Figure 7 | Effects of selection treatment (ambient- vs. drought-selected plants) on biomass ratio between root and shoot measured on individual plants after the drought event in the glasshouse.** Red points and error bars show means $\pm$ standard error of all individual plants. Grey points represent means for species (standard errors for species were not shown). Grey lines connected the same species between the selection treatments. Dashed lines represent statistically insignificant difference ($P > 0.05$) in trait values between the two selection treatments. The statistical tests for all species together were conducted by fitting block and selection treatment as fixed-effects terms, species composition and its interaction with selection treatment as random-effects terms in mixed-effects models. The statistical tests for each species were conducted by fitting block and selection treatment in general linear models. Source data are provided as a Source Data file. The numbers of pots per species pair and per selection treatment, and the results of statistical tests for each species separately and all species jointly are provided in the Source Data file.

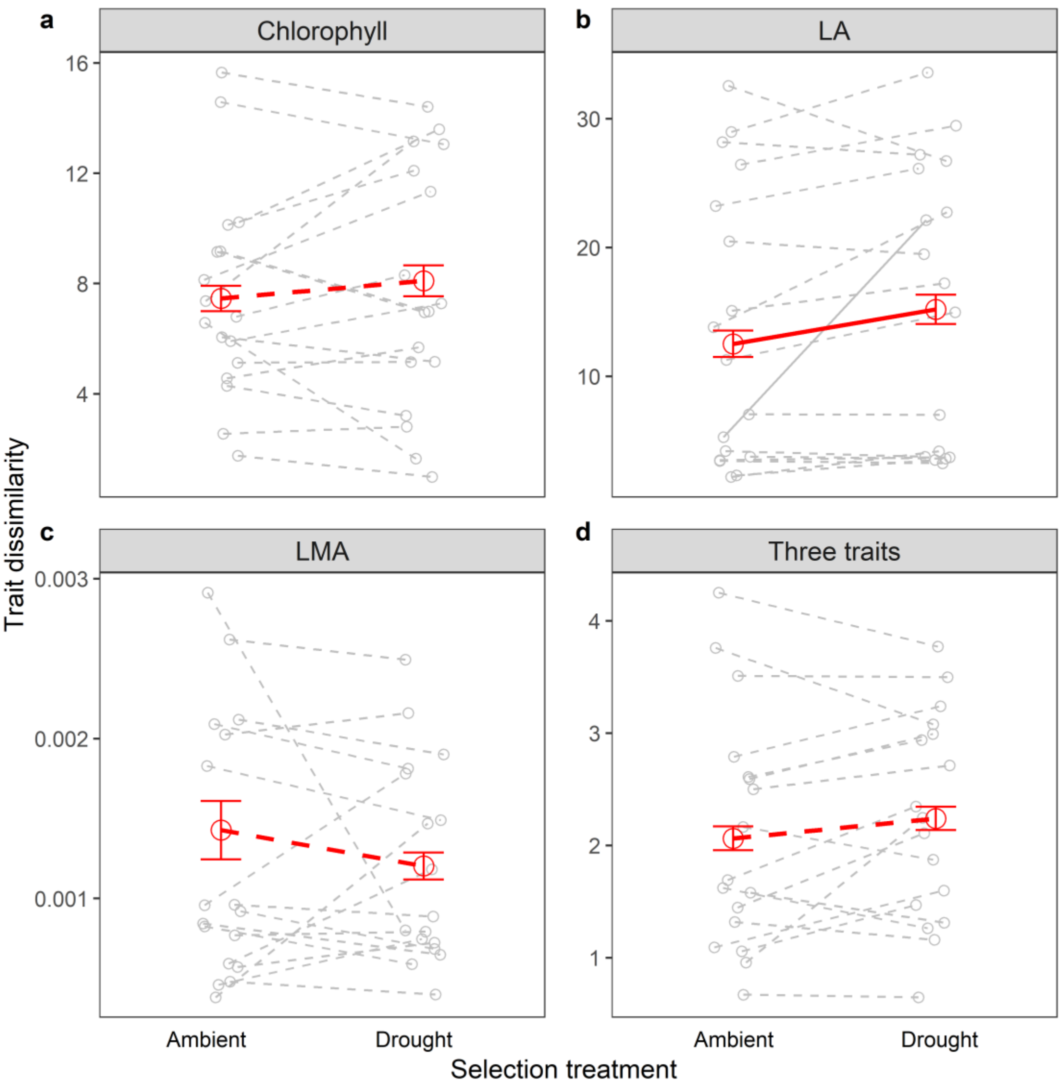


**Supplementary Figure 8 | Effects of selection treatment (ambient- vs. drought-selected plants) on trait dissimilarity between interacting species within 2-speices mixtures before the drought event in the glasshouse.** Trait dissimilarities were measured for leaf relative chlorophyll content (a), leaf area (b, LA), leaf mass per area (c, LMA) and the three traits together (d). Red points and error bars show means ± standard error of all mixtures. Grey points represent means for species pair (standard errors for species pairs were not shown). Grey lines connected the same species pair between the selection treatments. The solid red line indicates a marginally significant difference (averaged across species pairs) between the two selection treatments ($P <$ 0.10) from a mixed-effects model, in which block and selection treatment were set

fixed-effects terms, species composition and its interaction with selection treatment were set as random-effects terms (Supplementary Table 10). The solid grey line indicates a significant difference for a specific species pair between the two selection treatments ($P < 0.05$) from a general linear model, in which block and selection treatment were set as fixed-effects terms. Source data are provided as a Source Data file. The numbers of mixtures per species pair and per selection treatment, and the results of statistical tests for each species pair are provided in the Source Data file.

## REFERENCE

1 Roscher, C. *et al.* The role of biodiversity for element cycling and trophic interactions: an experimental approach in a grassland community. *Basic and Applied Ecology* **5**, 107-121, (2004).
