# Supplementary material for: Drought-exposure history increases complementarity between plant species in response to a subsequent drought
Source: Nat Commun. 2022 Jun 9;13:3217. doi: 10.1038/s41467-022-30954-9 (PMC9184649; doi:10.1038/s41467-022-30954-9)
Supplement: Supplementary file 3 — Description of Additional Supplementary Files [file 41467_2022_30954_MOESM3_ESM.docx]

**Description of Additional Supplementary Files**

**Supplementary Data 1:** Significance tests for the effects of species presence or absence on biodiversity effects on productivity.

**Supplementary Data 2:** Seed source information of each species in the Jena Experiment.

**Supplementary Data 3:** Significance tests for the effects of drought-selection history, plant origination from monoculture field plots and their interaction on biodiversity effects on productivity.

**Supplementary Data 4:** Significance tests for the effects of drought-selection history, functional group richness (as a linear term) history and their interaction on biodiversity effects on productivity.

**Supplementary Data 5:** Significance tests for the effects of drought-selection history, functional group richness (as a log-linear term) history and their interaction on biodiversity effects on productivity.
