# Supplementary material for: Drought-exposure history increases complementarity between plant species in response to a subsequent drought
Source: Nat Commun. 2022 Jun 9;13:3217. doi: 10.1038/s41467-022-30954-9 (PMC9184649; doi:10.1038/s41467-022-30954-9)
Supplement: Supplementary file 9 — Reporting Summary [file 41467_2022_30954_MOESM9_ESM.pdf]

nature portfolio

Corresponding author(s): Yuxin Chen, Bernhard Schmid

Last updated by author(s): Apr 25, 2022

Reporting Summary

Nature Portfolio wishes to improve the reproducibility of the work that we publish. This form provides structure for consistency and transparency in reporting. For further information on Nature Portfolio policies, see our Editorial Policies and the Editorial Policy Checklist.

Statistics

For all statistical analyses, confirm that the following items are present in the figure legend, table legend, main text, or Methods section.

n/a	Confirmed	
☐	☒	The exact sample size (n) for each experimental group/condition, given as a discrete number and unit of measurement
☐	☒	A statement on whether measurements were taken from distinct samples or whether the same sample was measured repeatedly
☐	☒	The statistical test(s) used AND whether they are one- or two-sided<br>*Only common tests should be described solely by name; describe more complex techniques in the Methods section.*
☐	☒	A description of all covariates tested
☐	☒	A description of any assumptions or corrections, such as tests of normality and adjustment for multiple comparisons
☐	☒	A full description of the statistical parameters including central tendency (e.g. means) or other basic estimates (e.g. regression coefficient) AND variation (e.g. standard deviation) or associated estimates of uncertainty (e.g. confidence intervals)
☐	☒	For null hypothesis testing, the test statistic (e.g. F, t, r) with confidence intervals, effect sizes, degrees of freedom and P value noted<br>*Give P values as exact values whenever suitable.*
☒	☐	For Bayesian analysis, information on the choice of priors and Markov chain Monte Carlo settings
☐	☒	For hierarchical and complex designs, identification of the appropriate level for tests and full reporting of outcomes
☐	☒	Estimates of effect sizes (e.g. Cohen's d, Pearson's r), indicating how they were calculated

Our web collection on statistics for biologists contains articles on many of the points above.

Software and code

Policy information about availability of computer code

Data collection	No software was used for data collection.
Data analysis	All analyses were conducted using the software R, version 3.6.3; mixed-effects models were fitted using the package ASReml for R, version 4.1.0.110. The code supporting the results are available at the Figshare digital repository (https://doi.org/10.6084/m9.figshare.14511060.v1).

For manuscripts utilizing custom algorithms or software that are central to the research but not yet described in published literature, software must be made available to editors and reviewers. We strongly encourage code deposition in a community repository (e.g. GitHub). See the Nature Portfolio guidelines for submitting code & software for further information.

Data

Policy information about availability of data

All manuscripts must include a data availability statement. This statement should provide the following information, where applicable:

- Accession codes, unique identifiers, or web links for publicly available datasets
- A description of any restrictions on data availability
- For clinical datasets or third party data, please ensure that the statement adheres to our policy

Source data are provided with this paper. The data supporting the findings of this study are available at the Figshare digital repository (https://doi.org/10.6084/m9.figshare.14511108.v1).

Field-specific reporting

Please select the one below that is the best fit for your research. If you are not sure, read the appropriate sections before making your selection.

☐ Life sciences ☐ Behavioural & social sciences ☒ Ecological, evolutionary & environmental sciences

For a reference copy of the document with all sections, see nature.com/documents/nr-reporting-summary-flat.pdf

Ecological, evolutionary & environmental sciences study design

All studies must disclose on these points even when the disclosure is negative.

Study description	To test whether an 8-year treatment of recurrent summer droughts would change biodiversity effects and species interactions of grassland plants when facing a new drought event, we grew ambient- vs. drought-selected plants of 12 species in a glasshouse. The plants were grown from seeds collected from 40 plots under 8-year treatments of yearly summer droughts vs. ambient precipitation in a biodiversity field experiment in Jena, Germany. Seed offspring of the 12 species were grown individually, in monocultures or in 2-species mixtures and subjected to a new drought event in the glasshouse.
Research sample	The 12 grassland species used in this study were from a field experiment in Jena, Germany, where the drought-exposure history occurred. They are common to the Central European Arrhenatherion grasslands.
Sampling strategy	The sample sizes were co-determined by seed availabilities in the field and germinations in the glasshouse. We obtained seeds of 17 species from the field, but only used 12 of them, because the other five species had either few seeds or low germination rates. The remaining 12 species had enough seedlings to manipulate both selection treatment (drought vs. ambient histories) and community treatment (mixture, monoculture and individual plant) at least in four replicated blocks. We established 958 pots: 257 pots of mixtures, 217 pots of monocultures and 484 pots of individual plants. For mixtures, there were 21 species pairs composed of 12 plant species from four functional groups. More detailed information on deciding species pairs are included in the Supplementary Methods section.
Data collection	Data were collected by Yuxin Chen, Anja Vogel, Cameron Wagg, Tianyang Xu, and Maitane Iturrate Garcia, with the help of trained helpers. Plant biomass was harvested with scissors three times: before, during and after an experimental drought event. Plant traits were measured with a SPAD-502 Plus chlorophyll meter (relative chlorophyll content), a LI-3100C Area Meter from LI-COR (leaf area), a Wescor vapor pressure osmometer VAPRO (osmometric pressure) and a SC-1 Leaf Porometer from Decagon Devices (leaf conductance). Data were recorded with laptops. More detailed information about data collection are available in the Methods section of the main text.
Timing and spatial scale	We transplanted the seedlings into pots from February 10–17 2017. After 14–16 weeks, when most of the species had reached peak aboveground biomass, we harvested all individuals in each pot (11×11×11.5 cm in size). The time span for the first harvest included both the time for trait measurements and for the immediately following biomass harvest. We completed the biomass harvest of each block within 1–2 days. After the first harvest of each block, plants were watered regularly and allowed to regrow until the 18th week from planting. This was followed by a second phase of two weeks without watering. At the end of the second phase, that is after 20 weeks from planting, we made a second aboveground harvest. During a third phase of seven weeks, pots were watered regularly again for recovery until most plants reached a new aboveground biomass peak again. At the end of the third phase, that is after 27 weeks from planting, we harvested both above- and belowground plant biomass. The final harvest took placed from August 21–23, 2017. This harvesting procedure mimics the common cutting management of the species in the field (and in comparable grasslands in the region).
Data exclusions	For the trait analyses with LA, LMA and the joint three traits as dependent variables, we removed one pot (B1P674) because the LA value of Alopecurus pratensis in this pot was extremely small (about 1/3 of the second minimum value of the same species in mixtures). All data exclusions are reported in the Methods section of the paper.
Reproducibility	The large number of replicate species compositions (21 species pairs) composed by a large number of replicate species (12 species common in central Europe) provides the confidence that the detected effects in this study can be generalized.
Randomization	We conducted the experiment blockwise. For each block, we randomly assigned different selection treatments and species compositions to pots tagged with random numbers. We randomized the spatial positions of pots within each block about once per week.
Blinding	The experimental treatments were randomized across pots. We randomized the spatial positions of pots within each block about once per week. Pots were identical in shape, size, color, and attached with tags identical in shape, size and color. These procedures blinded the researchers from the experimental treatments.

Did the study involve field work? ☐ Yes ☒ No

Reporting for specific materials, systems and methods

We require information from authors about some types of materials, experimental systems and methods used in many studies. Here, indicate whether each material, system or method listed is relevant to your study. If you are not sure if a list item applies to your research, read the appropriate section before selecting a response.

Materials & experimental systems

n/a	Involved in the study
☒	☐ Antibodies
☒	☐ Eukaryotic cell lines
☒	☐ Palaeontology and archaeology
☒	☐ Animals and other organisms
☒	☐ Human research participants
☒	☐ Clinical data
☒	☐ Dual use research of concern

Methods

n/a	Involved in the study
☒	☐ ChIP-seq
☒	☐ Flow cytometry
☒	☐ MRI-based neuroimaging
